# Pharmacokinetics and dosimetry of [^177^Lu]Lu-PSMA-617 and [^68^Ga]Ga-PSMA-11 in Japanese patients with PSMA-positive mCRPC

**DOI:** 10.1007/s12149-025-02079-8

**Published:** 2025-07-10

**Authors:** Shoko Takano, Anri Inaki, Kenji Hirata, Richard B. Sparks, Masahiko Sato, Satoshi Nomura, Toru Hattori, Hiroya Kambara, Quyen Nguyen, Tohru Shiga, Seigo Kinuya, Makoto Hosono

**Affiliations:** 1 Department of Radiation Oncology, Yokohama City University Graduate School of Medicine, 3-9 Fukuura, Kanazawa-ku, Yokohama, Kanagawa 236-0004 Japan; 2 Division of Functional Imaging, Exploratory Oncology Research and Clinical Trial Center (EPOC), National Cancer Center/Department of Nuclear Medicine, Kanazawa University, Kanazawa, Japan; 3 Department of Diagnostic Imaging, Graduate School of Medicine, Hokkaido University, Hokkaido, Japan; 4 CDE Dosimetry Services, Knoxville, TN USA; 5 PK Sciences, Novartis Pharma K.K., Tokyo, Japan; 6 Biostatistics Oncology, Novartis Pharma K.K., Tokyo, Japan; 7 Oncology Clinical Development, Novartis Pharma K.K., Tokyo, Japan; 8 PK Sciences Oncology, Novartis Pharma AG, Basel, Switzerland; 9 Department of Nuclear Medicine, Fukushima Medical University Hospital, Fukushima, Japan; 10 Department of Nuclear Medicine, Kanazawa University Hospital, Kanazawa, Japan; 11 Department of Radiology, Kindai University, Osaka, Japan

**Keywords:** [^177^Lu]Lu-PSMA-617, [^68^Ga]Ga-PSMA-11, Pharmacokinetics, Dosimetry, Metastatic castration-resistant prostate cancer, Radiation exposure

## Abstract

**Objective:**

This prospective, open-label, single-arm, phase 2 study evaluated the efficacy, safety, pharmacokinetics (PK) and dosimetry of [^177^Lu]Lu-PSMA-617 in Japanese patients with progressive PSMA+ mCRPC.

**Methods:**

This is a PK/dosimetry analysis of [^68^Ga]Ga-PSMA-11 and [^177^Lu]Lu-PSMA-617 in patients from Parts 1, 2, and 3 of the 4-part study. Blood and urine samples, serial PET/CT, planar, and SPECT/CT scans were collected post-administration of [^68^Ga]Ga-PSMA-11 (111–259 MBq) at screening and [^177^Lu]Lu-PSMA-617 (7.4 GBq ± 10%) during cycle 1. External radiation exposure in medical personnel and family members was measured once in each cycle from cycle 1 to 6, excluding the cycle where dosimetry was performed.

**Results:**

Of 35 patients included, 3 patients each had evaluable data for PK/dosimetry of [^68^Ga]Ga-PSMA-11 and [^177^Lu]Lu-PSMA-617. Both [^68^Ga]Ga-PSMA-11 and [^177^Lu]Lu-PSMA-617 showed a bi-exponential decline in blood concentrations post-dosage, with an initial rapid phase followed by a slower phase. For [^68^Ga]Ga-PSMA-11, terminal half-life (T_1/2_; geometric mean) was 3.93 h, total systemic clearance (CL) was 5.52 L/hr, and an apparent volume of distribution (V_z_) was 31.3 L. For [^177^Lu]Lu-PSMA-617, these values were 28.9 h, 1.71 L/hr, and 71.2 L, respectively. For [^68^Ga]Ga-PSMA-11 dosimetry, kidneys received the largest absorbed doses (0.23 ± 0.14 mGy/MBq), and effective dose was 0.030 mSv/MBq. For a full six-cycle cumulative injected activity of 44.4 GBq of [^177^Lu]Lu-PSMA-617, the lacrimal glands received the largest estimated absorbed dose of 90 ± 45 Gy. The mean absorbed dose to the kidneys (critical organ) was 0.34 Gy/GBq, resulting in a cumulative absorbed dose of 15 Gy for the full six-cycles. The radiation exposure was evaluated among 13 medical personnel, 8 who participated in administration, and family members. Measurements were taken at 8 sites including patients’ home. External radiation exposure to medical personnel and family members was minimal, with 0 μSv in 6/7 patients and 60 μSv in 1 patient.

**Conclusion:**

This is the first prospective Japanese study to demonstrate the use of [^68^Ga]Ga-PSMA-11 and [^177^Lu]Lu-PSMA-617 in patients with mCRPC. The absorbed doses in various organs for both radiopharmaceuticals were consistent with previously reported data. Minimal radiation exposure observed for medical personnel and caregivers highlights the safety of [^177^Lu]Lu-PSMA-617 during treatment, ensuring a secure treatment environment.

**Trial registration:**

This study is a prospective, open-label, multicenter, single-arm, phase 2 trial of [^177^Lu]Lu-PSMA-617 in patients with progressive PSMA + mCRPC in Japan (NCT05114746). The trial was initiated on 25-Jan-2022 (first patient first visit), with a primary analysis data cut-off on 8-Dec-2023. The study is ongoing. A total of 35 patients were screened and received [^68^Ga]Ga-PSMA-11, of whom 30 were included for efficacy and safety assessments of [^177^Lu]Lu-PSMA617 across Part 1 (safety run-in), Part 2 (post-taxane), and Part 3 (pre-taxane). Additionally, 3 patients each had evaluable data for PK and dosimetry assessments of [^68^Ga]Ga-PSMA-11 and [^177^Lu]Lu-PSMA-617. Informed consent was obtained from all participants before conducting any study-specific procedures.

**Supplementary Information:**

The online version contains supplementary material available at 10.1007/s12149-025-02079-8.

## Introduction

Prostate cancer, a significant global health concern, is the second most common cancer in men, the fifth leading cause of cancer-related death among men worldwide and the seventh leading cause of mortality in Japan [1, 2]. Despite the initial response rates, a considerable proportion develop castration-resistance and progress to metastatic castration-resistant prostate cancer (mCRPC) [3].

The current treatment paradigm for mCRPC includes androgen deprivation therapy (ADT) plus androgen receptor pathway inhibitor (ARPI) and taxane based chemotherapies including docetaxel and cabazitaxel which demonstrated clinical benefit in terms of radiographic progression-free survival (rPFS) and overall survival (OS) [4, 5]. The prostate-specific membrane antigen (PSMA) is a transmembrane glycoprotein, overexpressed in nearly all prostate cancers, making it an actionable theranostic target [6, 7]. Gallium (^68^Ga) gozetotide ([^68^Ga]Ga-PSMA-11) is an approved radiolabeled imaging agent in US and EU (not yet approved in Japan) with high diagnostic performance, aiding as patient selection agent for PSMA-targeted radioligand therapy (RLT) with lutetium ^177^Lu-vipivotide tetraxetan ([^177^Lu]Lu-PSMA-617) [8, 9]. [^177^Lu]Lu-PSMA-617 delivers β-particle radiation selectively to PSMA-positive (PSMA +) cells and the surrounding microenvironment [10, 11].

In the phase 3 VISION trial, [^177^Lu]Lu-PSMA-617 plus standard of care (SOC) demonstrated significant prolongation of rPFS and OS versus SOC alone in patients with PSMA+ mCRPC after progression on ARPI and taxane [12, 13]. In the phase 3 PSMAfore trial, [^177^Lu]Lu-PSMA-617 reduced the risk of rPFS by 59% versus a change in ARPI in patients with PSMA+ mCRPC who have progressed once on a previous ARPI in the pre-taxane setting [14]. For the clinical use of [^177^Lu]Lu-PSMA-617 as RLT, dosimetry studies are crucial to estimate the absorbed doses in both tumors and normal organs, providing strong evidence of safety and efficacy [15]. The VISION dosimetry sub-study in non-Japanese patients confirmed the safety of cumulative absorbed dose of 44.4 GBq of [^177^Lu]Lu-PSMA-617 administered over 6 cycles without inducing renal toxicity of grade 3 or higher Common Terminology Criteria for Adverse Events (CTCAE) [13].

Apart from assessing the safety, efficacy, pharmacokinetics (PK), and dosimetry of [^177^Lu]Lu-PSMA-617, it is crucial to evaluate the external dose rate of patients and potential risk of radioactive contamination for caregivers and relatives [16, 17]. Alleviating the anxiety of patients and their families regarding radiation exposure is essential for ensuring appropriate treatment. However, this is influenced by individual PK, the patient’s treatment situation, and the precautions for patients and their family. The International Atomic Energy Agency (IAEA) recommends keeping caregivers’ exposure below 5 mSv/year. In addition to multiple guidelines available, there are few reports on actual measured doses [18]. The guidance for administering [^177^Lu]Lu-PSMA-617 RLT vary between inpatient and outpatient settings, impacting the potential radiation exposure to caregivers, family members and healthcare professionals [19]. Therefore, ensuring the safety of everyone involved in RLT treatment is as crucial as ensuring patient safety.

This is the first prospective, Japanese study aimed to assess the PK and dosimetry of [^177^Lu]Lu-PSMA-617 and [^68^Ga]Ga-PSMA-11 in the same cohort of Japanese patients with mCRPC. The study also provides insights into airborne radioactivity concentration, the external radiation exposure experienced by medical personnel, family members, and in patients’ adjacent rooms during [^177^Lu]Lu-PSMA-617 treatment.

## Materials and methods

### Study design and eligibility criteria

This is a prospective, open-label, multicenter, single-arm, phase 2 study of [^177^Lu]Lu-PSMA-617 (NCT05114746) in the treatment of patients with progressive PSMA+ mCRPC in Japan. The study was initiated on 25-Jan-2022 (first patient first visit) and the data cut-off date of primary analysis was on 8-Dec-2023; the study is still ongoing.

The key eligibility criteria included patients with histological and/or cytological confirmation of prostate cancer, Eastern Cooperative Oncology Group (ECOG) performance status of 0–2, and a confirmed positive [^68^Ga]Ga-PSMA-11 positron emission tomography (PET)/computed tomography (CT) scan by the central assessment before initiating the [^177^Lu]Lu-PSMA-617 treatment (refer additional information in supplementary methods). Informed consent was collected from all eligible patients before conducting any study-specific procedures.

### Dosimetry and PK assessments

This was a 4-part study: Part 1 (safety run-in part), Part 2 (post-taxane part), Part 3 (pre-taxane part) and Part 4 (expansion part). In this current analysis (includes Part 1, 2 and 3), overall, 35 patients were planned for screening, and 28/35 patients derived from the initial 3 parts of the study were planned for [^177^Lu]Lu-PSMA-617 efficacy assessments. Efficacy results will be presented in a separate publication.

### ***Dosimetry and PK assessments for [***^***68***^***Ga]Ga-PSMA-11***

The [^68^Ga]Ga-PSMA-11 was administered as a single intravenous (IV) injection at a dosage of 111–259 MBq (3–7 mCi). The dosimetry and PK of [^68^Ga]Ga-PSMA-11 were optional assessments planned at the screening visit in Parts 1, 2, or 3, among 4–6 patients (regardless of the PSMA status) in selected sites. The dosimetry evaluation involved the PET/CT imaging data, along with blood and urine samples. Blood sample was collected at 0.083 h (h), 0.25, 0.5, 0.75, 1.42, 2.92, and 4.08 h post-injection. Urine was collected at the screening visit following [^68^Ga]Ga-PSMA-11 administration, between the end of injection and 1 h post-injection. PET/CT scans were scheduled at time intervals of 0, 0.5, 1, 2, and 4.25 h.

Regions of interest (ROIs) to create volume of interest (VOI) were constructed on the PET images for organs and tissues showing specific uptake of activity in various organs (refer additional information in supplementary methods) [20–22].

### ***Dosimetry and PK assessments for [***^***177***^***Lu]Lu-PSMA-617***

[^177^Lu]Lu-PSMA-617 IV infusion was administered at a dosage of 7.4 GBq ± 10% once every 6 weeks (± 1 week) for a maximum of 6 cycles. The dosimetry and PK assessments of [^177^Lu]Lu-PSMA-617 were mandatory for patients in Part 1 and optional in Parts 2 and 3 and planned in 4 to 6 PSMA+ patients at selected sites during cycle 1. Blood samples were collected at end of infusion (EOI), 0.33, 1, 2, 4, 24, 48, 72, and 120 h post-infusion. At specified times, 1 mL of blood was collected for radioactivity measurement by gamma counter. Urine aliquot assay data for [^177^Lu]Lu-PSMA-617, and total urine volume from start of injection to the first planar image collection time (1–2 h post-infusion) was collected.

The dosimetry evaluation necessitated whole body conjugate planar imaging data and 3D single-photon emission computed tomography (SPECT)/CT imaging, and blood and urine sampling during cycle 1. Serial gamma camera images (whole-body planar images) and 3D SPECT/CT scans of the upper abdomen (including the kidneys, liver, and spleen) were captured at 2, 24, 48, and 168 h post-infusion of [^177^Lu]Lu-PSMA-617 during cycle 1 (refer additional information in supplementary methods).

### Dose rate from patients

The dose rate (μSv/h) was measured using a semiconductor personal dosimeter (RPL Dosemeter, Chiyoda Technol Corporation, Japan) at a distance of 1 m from the patient’s body surface during cycle 1 at 1, 4, 24, 48, and 168 h post-infusion of [^177^Lu]Lu-PSMA-617 [23].

### External radiation exposure measurement

External radiation exposure measurements were mandated once in any cycle from 1 to 6, excluding the cycle where dosimetry was performed. These measurements were conducted for ≥ 1 participant at each site, varying based on the type of inpatient room where the patient was admitted (radiation treatment room or special measures room, which is a general hospital room with temporary radiation protection measures, as defined in the Medical Care Act in Japan). For further details on external radiation exposure in various scenarios, refer to supplementary methods section [23, 24].

### Statistical analysis

The PK analysis and dosimetry analysis included all patients who received at least one dosage of either [^177^Lu]Lu-PSMA-617 and [^68^Ga]Ga-PSMA-11 and had at least one evaluable PK or dosimetry data point. All participants who had any available data for external radiation exposure measurement were considered for analyses. All the analyses conducted in this study were descriptive in nature, focusing on the exploration and summarization of the data rather than hypothesis testing. This approach was chosen due to the complex and variable nature of the data, and the primary goal of understanding the behavior and effects of [^177^Lu]Lu-PSMA-617 and [^68^Ga]Ga-PSMA-11 in the patient population.

## Results

### Study population

Of the 40 patients screened, 35 were included; 5 were excluded due to screen failure. All 35 patients received at least one dosage of [^68^Ga]Ga-PSMA-11. In PK and dosimetry analysis, by the time of data cut-off, 3 patients each had evaluable dosimetry and PK data for [^68^Ga]Ga-PSMA-11 and for [^177^Lu]Lu-PSMA-617. All the 3 patients evaluated for [^68^Ga]Ga-PSMA-11 and the other 3 patients evaluated for [^177^Lu]Lu-PSMA-617 PK and dosimetry, respectively, were post-taxane population.

### ***PK of [***^***68***^***Ga]Ga-PSMA-11***

Blood concentrations (mean ± SD) of [^68^Ga]Ga-PSMA-11 were observed in all 3 patients at the time points of 0.083, 0.25, 0.5, 0.75, 1.4, 3, and 4 h post-dosage of [^68^Ga]Ga-PSMA-11 IV injection (Supplementary Table 2). The mean maximum concentration (C_max_ ± SD, percent injected activity per liter [%IA/L]) was 6.52 ± 1.15. The mean area under the concentration–time curve (AUC_last_ and AUC_inf_) were 9.17 hr*%IA/L and 19.7 hr*%IA/L, respectively (Table 1). Peak [^68^Ga]Ga-PSMA-11 whole blood concentrations were estimated at time zero (C_0_), except for a single patient for which the peak concentration was observed at 0.25 h post-dosage. A bi-exponential decline was observed in the blood concentrations, with a fast phase within the first 0.75 h and a slower phase up to 4.08 h post-dosage. The non-compartmental analysis demonstrated the geometric mean (mean ± SD) of total systemic clearance (CL) as 5.52 L/hr (5.96 ± 2.61) and an apparent volume of distribution (V_z_) as 31.3 L (31.6 ± 5.86). The geometric mean of terminal half-life was 3.93 h.

**Table 1:** Summary of [^68^Ga]Ga-PSMA-11 PK parameters (N = 3)

	C_max_ (%IA/L)	T_max_ (hr)	AUC_last_ (hr*%IA/L)	AUC_inf_ (hr*%IA/L)	CL (L/hr)	V_z_ (L)	T_1/2_ (hr)
Mean	6.52	NA	9.17	19.7	5.96	31.6	4.12
SD	1.15	NA	2.48	10.3	2.61	5.86	1.63
CV%	17.6	NA	27.0	52.2	43.8	18.5	39.7
Geometric mean	6.45	0.112	8.93	18.1	5.52	31.3	3.93
Geometric CV%	18.5	80.6	29.6	52.9	52.9	17.9	38.1

^*68*^*Ga* gallium-68, *AUC*_*inf*_ area under the curve to infinity, *AUC*_*last*_ area under the curve to the last measurable concentration, *CL* clearance, *C*_*max*_ maximum concentration, *CV* coefficient of variation, *hr* hour, *IA* injected activity, *L* liter, *MBq* megabecquerel, *PSMA* prostate specific membrane antigen, *T*_*1/2*_ half-life, *T*_*last*_ time to last measurable concentration, *T*_*max*_ time to maximum concentration, *V*_*z*_ volume of distribution

### ***PK of [***^***177***^***Lu]Lu-PSMA-617***

Blood concentrations (mean ± SD) of [^177^Lu]Lu-PSMA-617 were observed in all 3 patients from 0 h (immediately following the EOI of [^177^Lu]Lu-PSMA-617), 1, 2, 4, 24, 48, 72, and 120 h post-EOI (Supplementary Table 3), and the summary of PK parameters from whole blood is mentioned in Table 2. The original blood concentrations of [^177^Lu]Lu-PSMA-617 (kBq/mL) were converted to ng/mL. Peak [^177^Lu]Lu-PSMA-617 whole blood concentrations were observed at the EOI. Following T_max_, the blood concentrations followed a bi-exponential decline with a fast phase within the first 24–48 h and a slower phase up to 120 h post-EOI. The C_max_ was 13.5 ng/mL. The mean AUC_last_ and AUC_inf_ were 59.0 hr*ng/mL and 59.6 hr*ng/mL respectively. The geometric mean (mean ± SD) of total systemic clearance (CL) and apparent volume of distribution (V_z_) were 1.71 L/hr (1.72 ± 0.258) and 71.2 L (71.7 ± 10.6), respectively. It resulted in a T_1/2_ (geometric mean) of 28.9 h.

**Table 2:** Summary of [^177^Lu]Lu-PSMA-617 PK parameters (N = 3)

	C_max_ (ng/mL)	T_max_ (hr)	AUC_last_ (hr*ng/mL)	AUC_inf_ (hr*ng/mL)	CL (L/hr)	V_z_ (L)	T_1/2_ (hr)
Mean	13.5	NA	59.0	59.6	1.72	71.7	28.9
SD	3.06	NA	16.0	16.3	0.258	10.6	0.478
CV%	22.6	NA	27.2	27.4	15.0	14.7	1.65
Geometric mean	13.3	0.124	57.4	57.9	1.71	71.2	28.9
Geometric CV%	24.8	165	30.4	30.6	15.5	15.4	1.65

^*177*^*Lu* lutetium-177, *AUC*_*inf*_ area under the curve to infinity, *AUC*_*last*_ area under the curve to the last measurable concentration, *CL* clearance, *C*_*max*_ maximum concentration, *CV* coefficient of variation, *hr* hour, *L* liter; *min* minute, *MBq* megabecquerel, *mL* millilitre, *ng* nanogram, *PK* pharmacokinetics, *PSMA* prostate-specific membrane antigen, *SD* standard deviation, *T*_*1/2*_ half-life, *T*_*last*_ time to last measurable concentration, *T*_*max*_ time to maximum concentration, *V*_*z*_ volume of distribution

### ***Dosimetry of [***^***68***^***Ga]Ga-PSMA-11***

**Fig. 1:**
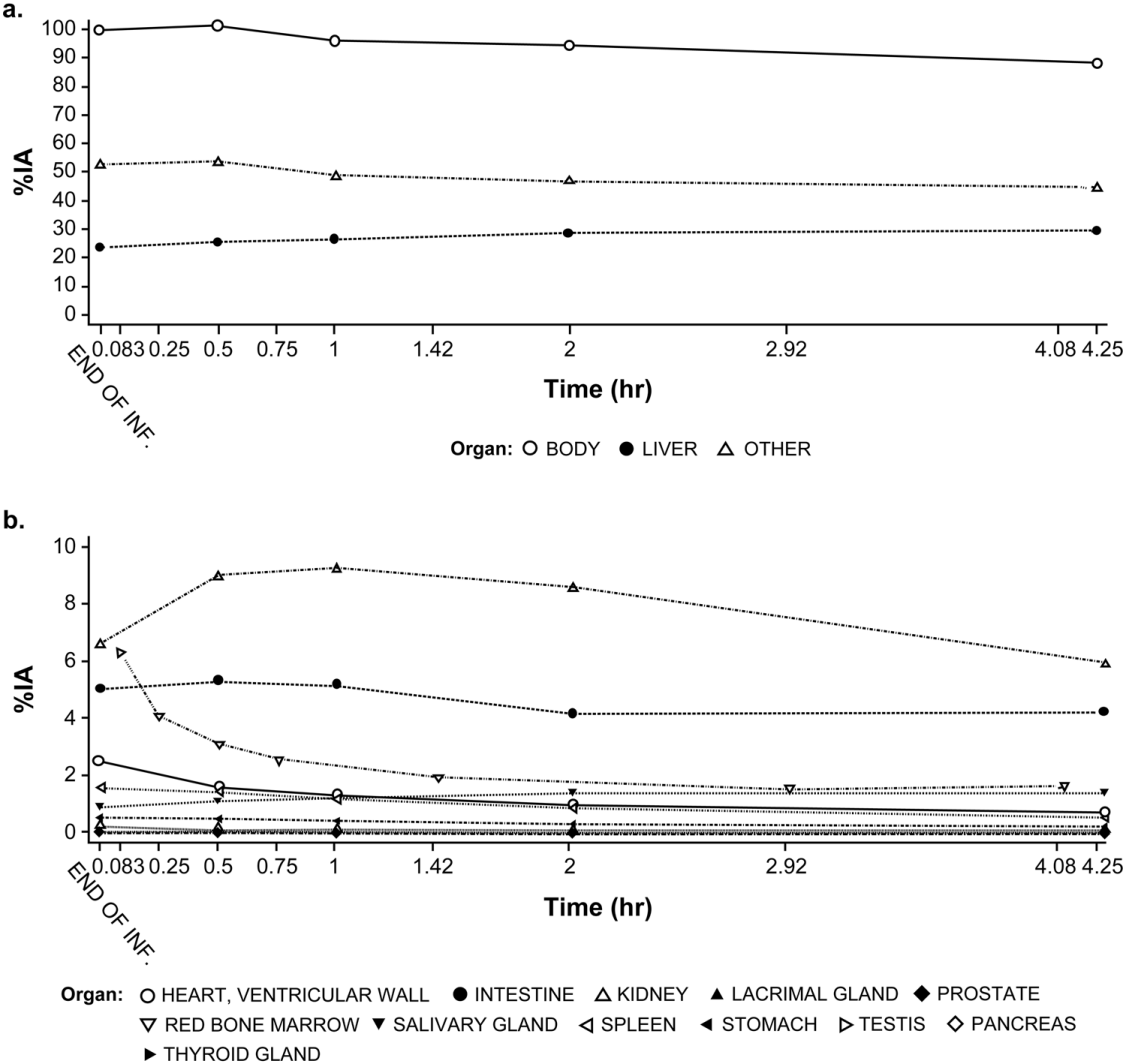
Mean percent injected activity-time (%IA) profile of [^68^Ga]Ga-PSMA-11: **a** Whole body, liver and other organs; **b** Heart, ventricular wall, intestine, kidney, lacrimal gland, prostate, red bone marrow, salivary gland, spleen, stomach, testis, pancreas, and thyroid gland. ^*68*^*Ga* gallium-68, *IA* injected activity, *PSMA* prostate-specific membrane antigen

The organ that showed the largest peak uptakes was the liver, ranging from 8.6% to 72% of the IA. After [^68^Ga]Ga-PSMA-11 administration, the organs receiving the largest absorbed doses (mean ± SD; mGy/MBq) were the kidneys (0.23 ± 0.14), the liver (0.17 ± 0.19), the lacrimal glands (0.13 ± 0.11), and the red marrow (0.017 ± 0.0023). The effective dose varied from 0.023 to 0.036 mSv/MBq with a mean ± SD of 0.030 ± 0.0070 mSv/MBq (Fig. 1a and b; Supplementary Table 4).

### ***Dosimetry of [***^***177***^***Lu]Lu-PSMA-617***

**Fig. 2:**
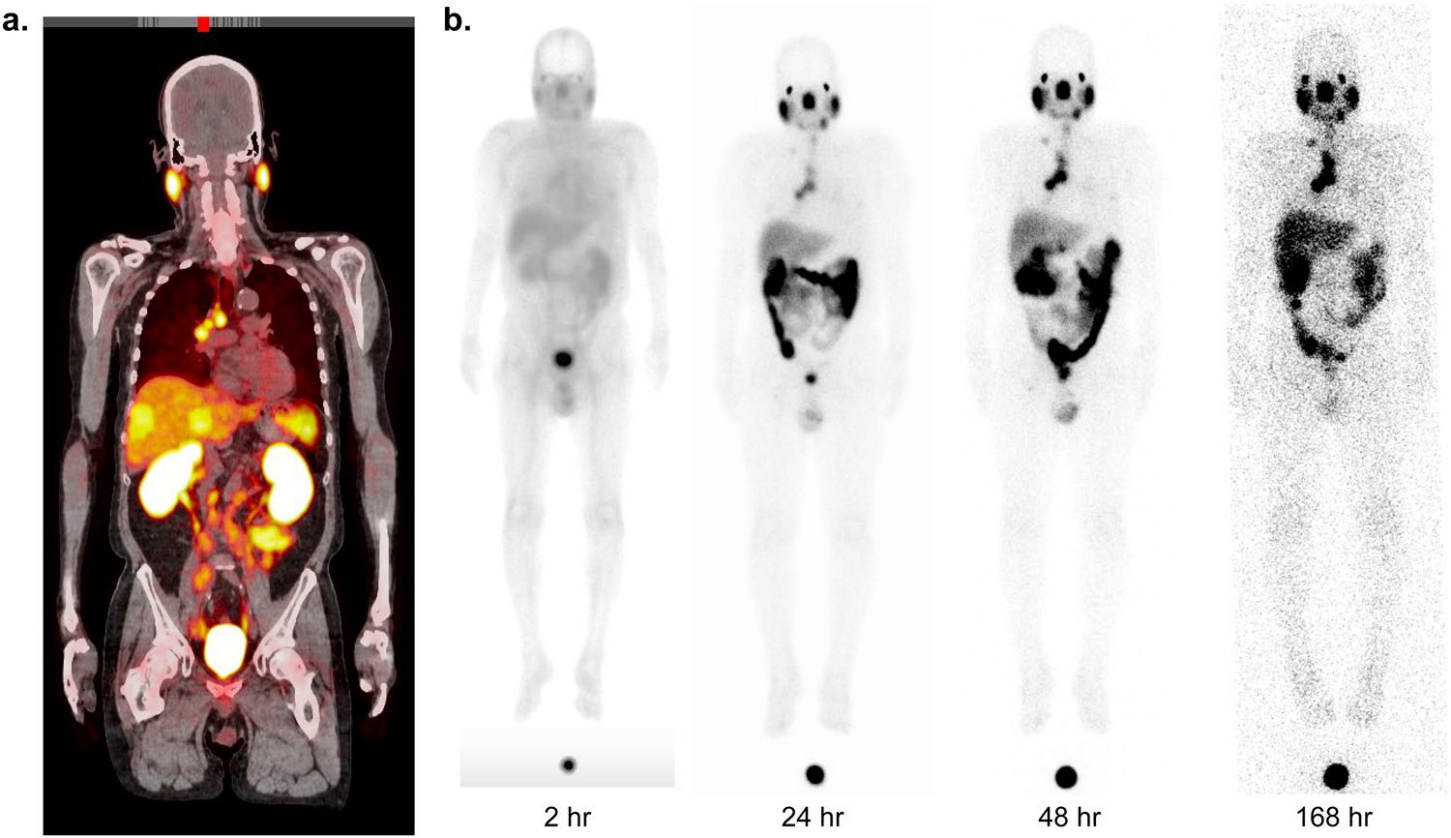
Representative images of [^68^Ga]Ga-PSMA-11 PET/CT scan (fusion image) at screening visit; **b** [^177^Lu]Lu-PSMA-617 planar images at four time points after [^177^Lu]Lu-PSMA-617 administration. This subject had tumors in the mediastinal lymph node, para-aortic lymph node, bone, and liver. ^*68*^*Ga* gallium-68, ^*177*^*Lu* lutetium-177, *CT* computed tomography, *hr* hour, *PET* positron emission tomography, *PSMA* prostate-specific membrane antigen

**Fig. 3:**
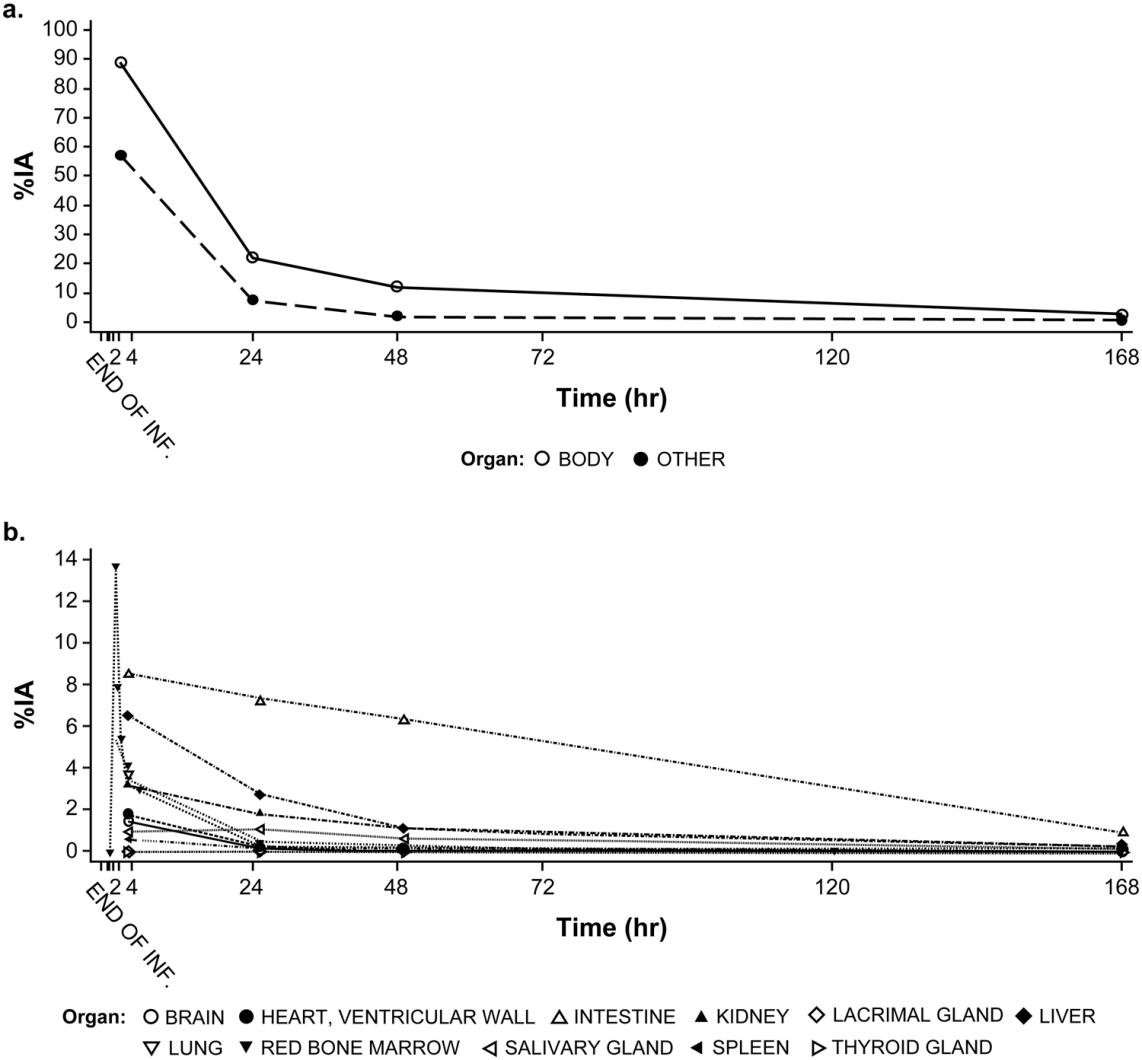
Mean percent injected activity-time (%IA) profile of [^177^Lu]Lu-PSMA-617: **a** Whole body and other organs; **b** Brain, heart, ventricular wall, intestine, kidney, lacrimal gland, liver, lung, red bone marrow, salivary gland, spleen, and thyroid gland. ^*177*^*Lu* lutetium-177, *EOI* end of infusion, *IA* injected activity, *PSMA* prostate-specific membrane antigen

Accumulation of [^177^Lu]Lu-PSMA-617 was visualized in the same lesion as [^68^Ga]Ga-PSMA-11 uptake in normal organs and tumors (Fig. 2a and b). The %IA (mean ± SD) in the whole body decreased over time, with values of 89% ± 9.4%, 22% ± 4.5%, 12% ± 3.5% and 2.8% ± 1.1% at 2, 24, 48, and 168 h post-dosage, respectively. The organs that showed the largest peak uptakes were the GI tract ranging from 7.8% to 11% of the IA from the first to third time points, and red marrow ranging from 12 to 14% of the IA at first time point. After [^177^Lu]Lu-PSMA-617 administration, the lacrimal glands had the largest absorbed dose (mean ± SD: 2.0 ± 1.0 Gy/GBq), followed by the salivary glands (0.78 ± 0.026 Gy/GBq). After a full 6-cycle cumulative IA of 44.4 GBq, the estimated absorbed doses (mean ± SD) to the lacrimal glands and salivary glands were 90 ± 45 and 35 ± 1.2 Gy, respectively. For the critical organs, the mean absorbed dose to the kidneys was 0.34 ± 0.059 Gy/GBq, which resulted in a cumulative absorbed dose of 15 ± 2.6 Gy for full 6 cycles. The red marrow absorbed a dose of 0.033 ± 0.0072 Gy/GBq, with an estimated full 6-cycle absorbed dose of 1.5 ± 0.32 Gy (Fig. 3a and b; Supplementary Table 5).

### ***Dose rate of [***^***177***^***Lu]Lu-PSMA-617***

Dose rate measured at a point 1 m from the surface of the patient administered with [^177^Lu]Lu-PSMA-617, peaked at 1 h post-dosage (29.6 to 37.8 µSv/h) and then decreased over time (18.9 to 26.8 μSv/h at 4 h, 4.4 to 8.55 μSv/h at 24 h, 3.27 to 4.5 μSv/h at 48 h, and 0 to 1.07 μSv/h at 168 h post-dosage).

### ***External radiation exposure to the medical personnel from preparation before administration to completion of [***^***177***^***Lu]Lu-PSMA-617 administration***

The radiation exposure was evaluated in 13 medical personnel (with 1 or 2 medical personnel per patient) of 8 patients. These measurements were conducted at 8 sites (radiation treatment room: 4 sites and special measures rooms: 4 sites). In 7 individuals who wore radiation protection suits, the maximum exposure was 4 μSv inside the suit; the average external exposure (mean ± SD) was 9.85 μSv (± 16.832) (Table 3). Except for one outlier (63.6 μSv), the exposure was limited (for more information, refer Supplementary results).

**Table 3:** External radiation exposure to medical personnel while administering [^177^Lu]Lu-PSMA-617

Type of external exposure to medical personnel	Inside radiation protection suit [μSv]	Outside radiation protection suit [μSv]	Exposure time spent [min]
From preparation to completion of [^177^Lu]Lu-PSMA-617 administration			
n	7	13	13
Mean (SD)	2.13 (1.641)	9.85 (16.832)	84.5 (70.81)
While accompanying the patient			
n	4	4	4
Mean (SD)	0.23 (0.126)	0.38 (0.050)	4.5 (1.29)

^*177*^*Lu* lutetium-177, *μSv* microsievert, *min* minute, *PSMA* prostate-specific membrane antigen, *SD* standard deviation

### Exposure to the medical personnel accompanying the patient

For 4 patients who received [^177^Lu]Lu-PSMA-617, the dose was recorded while 4 medical personnel escorted the patient from the nuclear medicine examination room, where [^177^Lu]Lu-PSMA-617 was administered, to the inpatient room (special measures room). The maximum time accompanied with patients (n = 4) in a hospital room was 6 min, and the dose exposure during this process was extremely limited, regardless of whether a radiation protection suit was worn (Table 3).

### Exposure to the medical personnel when entering a hospital room

**Fig. 4:**
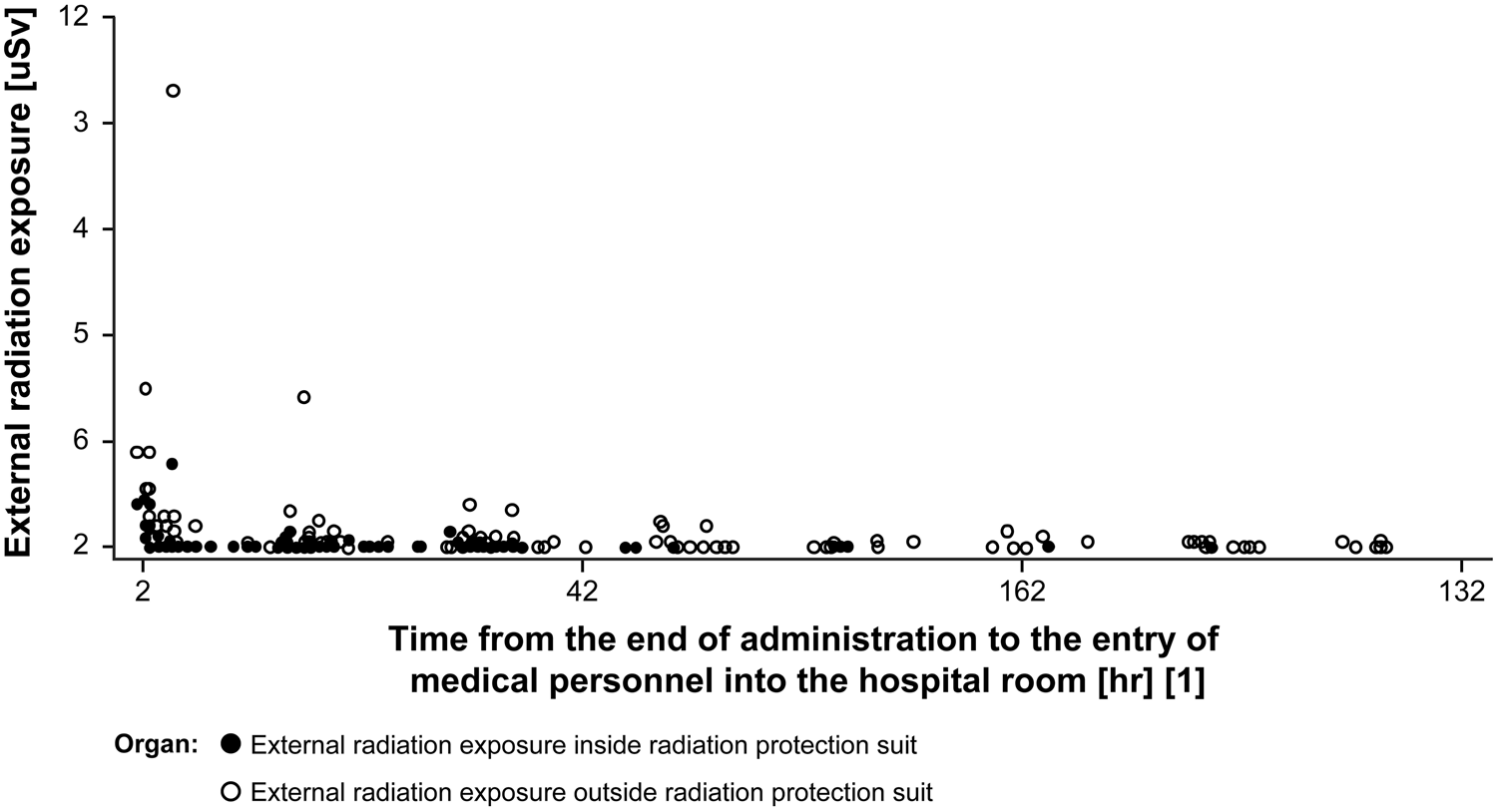
Plot of external radiation exposure of medical personnel when entering a hospital room. ^a^Time from the end of administration to the entry of medical personnel into the hospital room = Entry time of medical personnel into the hospital room − Administration end time. *μSv* microsievert

Radiation doses were measured for 170 instances of medical personnel entering and exiting the room of 8 patients (Fig. 4). Exposure doses were minimal, regardless of whether a radiation protection suit was worn.

### External radiation dose rate in the patient’s inpatient room

**Fig. 5:**
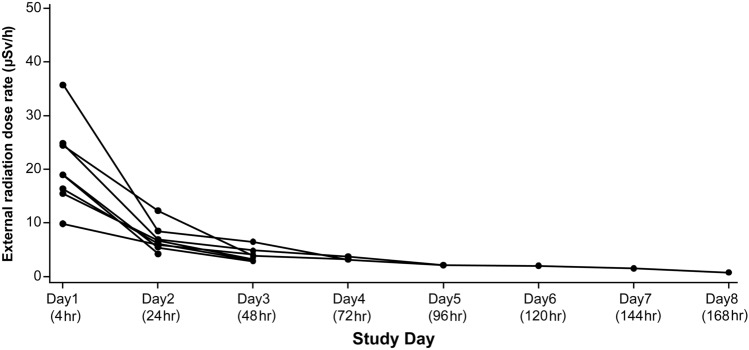
Plot of external radiation dose rate in patient's hospital room. If a patient is measured in multiple cycles, all measurements are used for the plot. Each line is constructed by dose rate measured within same cycle. *μSv* microsievert, *hr* hour

The external radiation exposure in the inpatient room was measured for 8 patients (4 patients each in radiation treatment room and special measures room), including one patient in the radiation treatment room during both the first and second cycles. The dose rate from a distance of 1 m from the patient decreased over time (Fig. 5; Supplementary Table 7).

### External radiation dose rate in the adjacent room of patients’ room

This was measured in 2 patients. The dose rates were 0 μSv/h (7.5 m) to 1.5 μSv/h (4.5 m) at 4 h post-dosage, 0 μSv/h (7.5 m) to 0.3 μSv/h (4.5 m) at 24 h post-dosage, and 0.3 μSv/h (4.5 m) at 48 h post-dosage (n = 1 for 48 h, n = 2 for 4 and 24 h).

### Airborne radioactivity concentration in the patient’s inpatient room

Airborne radioactivity concentrations were measured in 9 patients (radiation treatment room: 5 patients, special measures room: 4 patients). After administration of [^177^Lu]Lu-PSMA-617, the airborne radioactivity concentrations were below the limit of detection (1.1 × 10^−6^ Bq/cm^3^ or 9.5 × 10^−7^ Bq/cm^3^) in all 9 patients.

### Measurement of external radiation exposure to family members living with the patient

#### Exposure of the person accompanying the patient home

For 7 patients’ family members, the external radiation exposure when accompanied during journey while returning home from the hospital was measured. The mean ± SD was 1.37 ± 0.822 μSv in family members relatively close to the study site (maximum accompanying time: 60 min) (Table 4). The exposure dose was minimal even when the patient was immediately discharged from the hospital and accompanied home.

**Table 4:** External radiation exposure to person accompanying the patient

External radiation exposure of the person accompanying the participant until the participant goes home [μSv]	
n	7
Mean (SD)	1.37 (0.822)
Median	1.40
Q1–Q3	0.60–2.20
Min–Max	0.5–2.6
Time required for subject to return home/visit [min]	
n	7
Mean (SD)	43.3 (18.41)
Median	45.0
Q1–Q3	20.0–60.0
Min–Max	18–60
Family member radiation exposure [mSv]	
n	7
Mean (SD)	0.009 (0.0227)
Median	0.000
Q1–Q3	0.000–0.000
Min–Max	0.00–0.06
Days from administration date to hospital discharge date [day]	
n	7
Mean (SD)	4.3 (1.98)
Median	4.0
Q1–Q3	3.0–7.0
Min–Max	2–7
The length of time the family member approaches the participant (< 1 m) [min]	
n	72
Mean (SD)	204.1 (255.44)
Median	60.0
Q1–Q3	30.0–300.0
Min–Max	3–1080

#### Family member radiation exposure

The radiation exposure to family members was less than the lower limit of quantification (< 50 μSv) [23] in 6 of 7 patients and 60 μSv except in 1 patient (for more information refer supplementary results). The median time of proximity to the patient within 1 m/d was 60 min, indicating that many family members avoided being close to the patient for several days after discharge (Table 4) and that patients and their family members were living at home after understanding the explanation.

## Discussion

This prospective study provides PK and dosimetry data of [^68^Ga]Ga-PSMA-11 and [^177^Lu]Lu-PSMA-617 in Japanese patients with PSMA+ mCRPC previously treated with ARPI and taxane therapy. Additionally, this study offers a comprehensive understanding on the external radiation exposure data from patients, the impact of [^177^Lu]Lu-PSMA-617 on individuals accompanying the patient, and information on radiation exposure to third parties, including public.

The PK analysis revealed that both [^68^Ga]Ga-PSMA-11 and [^177^Lu]Lu-PSMA-617 were noted in all patients’ blood concentrations, with different decay rates and terminal half-lives. Following IV administrations of [^68^Ga]Ga-PSMA-11 and [^177^Lu]Lu-PSMA-617, the blood concentrations showed a bi-exponential decline, with a rapid phase within the first 0.75 h and a slower phase up to 4.08 h post-dosage for [^68^Ga]Ga-PSMA-11, and with a rapid phase within the first 24 to 48 h and a slower phase up to 120 h post-dosage for [^177^Lu]Lu-PSMA-617.

For [^68^Ga]Ga-PSMA-11 dosimetry, the organs receiving the largest absorbed doses (mGy/MBq) were kidneys (0.23), followed by liver (0.17), lacrimal glands (0.13), and the red marrow (0.017). These data were consistent with earlier studies [25, 26]. Sandgren et al. reported doses of 0.24, 0.053, 0.11, and 0.015 mGy/MBq in the kidneys, liver, lacrimal glands, and red marrow, respectively, with [^68^Ga]Ga-PSMA-11. The effective dose of [^68^Ga]Ga-PSMA-11 was 0.022 mSv/MBq, with the kidneys and lacrimal glands receiving the highest organ doses [25]. Similarly, another study noted absorbed doses for [^68^Ga]Ga-PSMA-11 of 0.2620, 0.0309, and 0.0092 mGy/MBq in the kidneys, liver, and red marrow, respectively [26]. Moreover, the [^68^Ga]Ga-PSMA-11 label indicates mean doses of 0.3714, 0.0409, and 0.0114 mGy/MBq in the kidneys, liver, and red marrow [27]. These studies demonstrated that absorbed doses are relatively consistent across the kidneys, lacrimal glands, and red marrow; however, the absorbed doses in the liver show some variation. The differences in the liver absorbed dose in the present study and in the Sandgren et al. study and other studies were attributed to an outlier patient with unusually large and atypical liver uptake [25, 26]. Excluding the outlier, the average liver absorbed dose for the two remaining patients is 0.055 mGy/MBq, aligning with the findings of Sandgren et al. study and showing no significant difference from the other studies.

For [^177^Lu]Lu-PSMA-617 dosimetry, the lacrimal glands received highest absorbed dose (2.0 Gy/GBq), followed by the salivary glands (0.78 Gy/GBq). The difference in the absorbed does between [^68^Ga]Ga-PSMA-11 and [^177^Lu]Lu-PSMA-617 in the lacrimal glands can be attributed to several factors, including the longer physical and biological half-lives of [^177^Lu]Lu-PSMA-617, resulting in a greater number of decays and higher absorbed energy, among other contributing factors. The kidneys absorbed a dose of 0.34 Gy/GBq, resulting in an estimated cumulative absorbed dose of 15 Gy after a full 6-cycles (44.4 GBq), which is within the conventional acceptable limit of 23 Gy and European Association of Nuclear Medicine (EANM) guideline of 40 Gy [28, 29]. As the kidney absorbed dose remains within these safety limits, the lacrimal glands and salivary glands appear to be the primary sites of accumulation for these compounds, suggesting a higher risk of potential toxicity and adverse events in these organs.

The dosimetry estimates of [^177^Lu]Lu-PSMA-617 were comparable between VISION sub-study and the present study. Conducted at 4 sites across Germany, the VISION dosimetry sub-study quantified absorbed doses of [^177^Lu]Lu-PSMA-617 in the kidneys and other organs, reporting an absorbed dose of 2.10 ± 0.47 Gy/GBq in lacrimal glands, which is comparable to the 2.0 ± 1.0 Gy/GBq in the present study. Similarly, PK parameters were also consistent between the two studies. Overall, there appears to be no apparent difference in PK between the current Japanese study and the VISION sub-study [13].

The International Commission on Radiological Protection (ICRP) recommends that radiation exposure for medical staff should be < 100 mSv in 5 years and 50 mSv in any single year, and the public exposure be kept below 1 mSv per year [30]. To adhere to these limitations, the Australian radiation protection and nuclear safety agency (ARPANSA) study recommend that external exposure should not exceed 25 μSv/h at a distance of 1 m from patient at the time of discharge [31]. The discharge criteria for clinical trials set in accordance with Japanese regulations were met: the patient was discharged once the radiation level fell below 5 μSv/h. By adhering to special behavioral restrictions (supplementary methods) to minimize exposure, the radiation exposure was kept to a minimum. These findings underscore the safety of these treatments for both medical personnel and family members. Notably, this study is the first to evaluate radiation exposure doses for both medical personnel and patients’ families, addressing a gap in previous research. The present study did not assess the cumulative radiation exposure for medical personnel including health care professionals resulting from their daily medical practice. The exposure measurements provided are based on single instances and do not reflect the potential cumulative exposure that medical personnel may experience over time. The radiation exposure to family members was minimal and, caution is needed when interpreting very low radiation exposure.

The study has some limitations, limited sample size and variation in the behavioral guidelines followed by patients’ families to minimize radiation exposure.

## Conclusion

This first prospective Japanese study provides valuable insights into the PK, dosimetry, and radiation exposure associated with [^68^Ga]Ga-PSMA-11 imaging and [^177^Lu]Lu-PSMA-617 treatment in Japanese patients with mCRPC. Pharmacokinetics and dosimetry results are comparable to previous studies including the VISION sub-study and the external radiation exposure data could inform the safe and effective use of these radiopharmaceuticals in clinical practice. Further research with a larger, more diverse sample, a control group, and various methods for measuring radiation exposure would further strengthen and validate these findings.

## Supplementary Information

Below is the link to the electronic supplementary material.

Supplementary file1 (DOCX 117 KB)

## Data Availability

Novartis is committed to sharing with qualified external researchers, access to patient-level data, and supporting clinical documents from eligible studies. These requests are reviewed and approved by an independent review panel based on the scientific merit. All data provided are anonymized to respect the privacy of patients who have participated in the trial in line with applicable laws and regulations. This trial data availability is according to the criteria and process described on https://www.clinicalstudydatarequest.com.

## References

[1] Cancer Information Service, National Cancer Center, Japan (Vital Statistics of Japan, Ministry of Health, Labour and Welfare) (n.d.) Cancer Statistics. https://ganjoho.jp/reg_stat/statistics/data/dl/excel/cancer_mortality(1979-2022)H27.xls. Accessed 19-02-2025.

[2] Rawla P. Epidemiology of prostate cancer. World J Oncol. 2019;10:63–89. 31068988 10.14740/wjon1191 PMC6497009

[3] Nussbaum N, George DJ, Abernethy AP, Dolan CM, Oestreicher N, et al. Patient experience in the treatment of metastatic castration-resistant prostate cancer: state of the science. Prost Cancer Prostatic Dis. 2016;19:111–21 (**[PMID: 26832363]**). 10.1038/pcan.2015.42 PMC4868871 26832363

[4] Ryan CJ, Smith MR, De Bono JS, Molena A, Logothetis CJ, et al. Abiraterone in metastatic prostate cancer without previous chemotherapy. N Eng J Med. 2013;368:138–48 (**[PMID: 23228172]**). 10.1056/NEJMoa1209096 PMC3683570 23228172

[5] Turco F, Gillessen S, Cathomas R, Buttigliero C, Vogl UM. Treatment landscape for patients with castration-resistant prostate cancer: Patient selection and unmet clinical needs. Res Rep Urol. 2022;14:339–50 (**[PMID: 36199275]**). 36199275 10.2147/RRU.S360444 PMC9529226

[6] Ghosh A, Heston WDW. Tumor target prostate specific membrane antigen (PSMA) and its regulation in prostate cancer. J Cell Biochem. 2004;91:528–39 (**[PMID: 14755683]**). 14755683 10.1002/jcb.10661

[7] Mannweiler S, Amersdorfer P, Trajanoski S, Terrett JA, King D, et al. Heterogeneity of prostate-specific membrane antigen (PSMA) expression in prostate carcinoma with distant metastasis. Pathol Oncol Res. 2009;15:167–72 (**[PMID: 18802790]**). 18802790 10.1007/s12253-008-9104-2

[8] Fendler WP, Eiber M, Beheshti M, Bomanji J, Ceci F, et al. ^68^Ga-PSMA PET/CT: joint EANM and SNMMI procedure guideline for prostate cancer imaging: version 1.0. Eur J Nucl Med Mol Imaging. 2017;44:1014–24 (**[PMID: 28283702]**). 28283702 10.1007/s00259-017-3670-z

[9] Jadvar H, Calais J, Fanti S, Feng F, Greene KL, et al. Appropriate use criteria for prostate-specific membrane antigen PET imaging. J Nucl Med. 2022;63:59–68 (**[PMID: 34593595]**). 34593595 10.2967/jnumed.121.263262 PMC8717184

[10] Benešová M, Schäfer M, Bauder-Wüst U, Afshar-Oromieh A, Kratochwil C, et al. Preclinical evaluation of a tailor-made DOTA-conjugated PSMA inhibitor with optimized linker moiety for imaging and endoradiotherapy of prostate cancer. J Nucl Med. 2015;56:914–20 (**[PMID: 25883127]**). 25883127 10.2967/jnumed.114.147413

[11] Emmett L, Willowson K, Violet J, Shin J, Blanksby A, et al. Lutetium 177 PSMA radionuclide therapy for men with prostate cancer: a review of the current literature and discussion of practical aspects of therapy. J Med Rad Sci. 2017;64:52–60 (**[PMID: 28303694]**). 10.1002/jmrs.227 PMC5355374 28303694

[12] Sartor O, Bono Jd, Chi KN, Fizazi K, Herrmann K, et al. Lutetium-177–PSMA-617 for metastatic castration-resistant prostate cancer. N Eng J Med. 2021;385:1091–103 (**[PMID: 34161051]**). 10.1056/NEJMoa2107322 PMC8446332 34161051

[13] Herrmann K, Rahbar K, Eiber M, Sparks R, Baca N, et al. Renal and multiorgan safety of ^177^Lu-PSMA-617 in patients with metastatic castration-resistant prostate cancer in the VISION dosimetry substudy. J Nucl Med. 2024;65:71–8 (**[PMID: 38050121]**). 38050121 10.2967/jnumed.123.265448 PMC10755516

[14] Sartor O, Gauna DEC, Herrmann K, de Bono JS, Shore ND, et al. LBA13 phase III trial of [^177^Lu]Lu-PSMA-617 in taxane-naive patients with metastatic castration-resistant prostate cancer (PSMAfore). Ann Oncol. 2023;34:S1324–5.

[15] Li L, Wang J, Wang G, Wang R, Jin W, et al. Comparison of novel PSMA-targeting [^177^Lu]Lu-P17–087 with its albumin binding derivative [^177^Lu]Lu-P17–088 in metastatic castration-resistant prostate cancer patients: a first-in-human study. Eur J Nucl Med Mol Imaging. 2024;51:2794–805 (**[PMID: 38658392]**). 38658392 10.1007/s00259-024-06721-x

[16] Stefanoyiannis AP, Ioannidou SP, Round WH, Carinou E, Mavros MN, et al. Radiation exposure to caregivers from patients undergoing common radionuclide therapies: a review. Radiat Prot Dosimet. 2015;167:542–51 (**[PMID: 25431487]**). 10.1093/rpd/ncu338 25431487

[17] de Bakker M, Dominicus N, Meeuwis A, Janssen M, Konijnenberg MW, et al. Urinary excretion kinetics of [^177^Lu]Lu-PSMA-617. Eur J Nucl Med Mol Imaging. 2023;50:3572–5 (**[PMID: 37421427]**). 37421427 10.1007/s00259-023-06328-8 PMC10547615

[18] Phillipe J, Calais ACP, Harvey Turner J. Management of fear of radiation exposure in carers of outpatients treated with iodine-131. Ann Nucl Med. 2012;26:230–7 (**[PMID: 22610385]**). 10.1007/s12149-012-0603-6 22610385

[19] Vorster M, Warwick J, Lawal I, Toit PD, Vangu M, et al. South African guidelines for receptor radioligand therapy (RLT) with Lu-177-PSMA in prostate cancer. S Afr J Surg. 2019;57:45–51 (**[PMID: 31773936]**). 31773936

[20] Siegel JA, Thomas SR, Stubbs JB, Stabin MG, Hays MT, et al. MIRD pamphlet no. 16: Techniques for quantitative radiopharmaceutical biodistribution data acquisition and analysis for use in human radiation dose estimates. J Nucl Med. 1999;40:37S-61S (**[PMID: 10025848]**). 10025848

[21] Stabin MG, Sparks RB, Crowe E. 2005 OLINDA/EXM: the second-generation personal computer software for internal dose assessment in nuclear medicine. J of Nucl Med. 2005;46:1023–7. 15937315

[22] Stabin MG, Farmer A. OLINDA/EXM 2.0: the new generation dosimetry modeling code. J Nucl Med. 2012;53:585.

[23] RPL Dosemeter for Individual Monitoring. https://www.c-technol.co.jp/en/pdf/Personal-Dosimetry-System_5th_Edition.pdf. Accessed 19-02-2025, 2025.

[24] Yamamoto H, Kishida M. Radiation monitoring of the workplace (VI). Radioisotopes. 1977;26:432–42 (**[PMID: 578968]**). 578968 10.3769/radioisotopes.26.6_432

[25] Sandgren K, Johansson L, Axelsson J, Jonsson J, Ögren M, et al. Radiation dosimetry of [^68^Ga]PSMA-11 in low-risk prostate cancer patients. EJNMMI Phys. 2019;6:11 (**[PMID: 30631980]**). 30631980 10.1186/s40658-018-0239-2 PMC6328430

[26] Afshar-Oromieh A, Hetzheim H, Kübler W, Kratochwil C, Giesel FL, et al. Radiation dosimetry of ^68^Ga-PSMA-11 (HBED-CC) and preliminary evaluation of optimal imaging timing. Eur J Nucl Med Mole Imaging. 2016;43:1611–20 (**[PMID: 27260521]**). 10.1007/s00259-016-3419-0 27260521

[27] LOCAMETZ® (kit for the preparation of gallium Ga 68 gozetotide injection), for intravenous use Initial U.S. Approval: 2020. 2020; https://www.accessdata.fda.gov/drugsatfda_docs/label/2022/215841s000lbl.pdf. Accessed 19–02–2025, 2025.

[28] Emami B, Lyman J, Brown A, Coia L, Goitein M, et al. Tolerance of normal tissue to therapeutic irradiation. Int J Radiat Oncol Biol Phys. 1991;21:109–22 (**[PMID: 2032882]**). 2032882 10.1016/0360-3016(91)90171-y

[29] Gleisner KS, Chouin N, Gabina PM, Cicone F, Gneisin S, et al. EANM dosimetry committee recommendations for dosimetry of ^177^Lu-labelled somatostatin-receptor- and PSMA-targeting ligands. Eur J Nucl Med Mol Imaging. 2022;49:1778–809 (**[PMID: 35284969]**). 35284969 10.1007/s00259-022-05727-7 PMC9015994

[30] ICRP Publication 103: The 2007 Recommendations of the International Commission on Radiological Protection. https://www.icrp.org/docs/icrp_publication_103-annals_of_the_icrp_37(2-4)-free_extract.pdf. Accessed 19–02–2025, 2025. 10.1016/j.icrp.2007.10.003 18082557

[31] ARPANSA Recommendations. Discharge of Patients Undergoing Treatment with Radioactive Substances Radiation Protection Series Publication No.4. https://www.arpansa.gov.au/sites/default/files/legacy/pubs/rps/rps4.pdf. Accessed 19–02–2025, 2025.

