# Supplementary material for: Pharmacokinetics and dosimetry of [177Lu]Lu-PSMA-617 and [68Ga]Ga-PSMA-11 in Japanese patients with PSMA-positive mCRPC
Source: Ann Nucl Med. 2025 Jul 10;39(11):1201–12. doi: 10.1007/s12149-025-02079-8 (PMC12559119; doi:10.1007/s12149-025-02079-8)
Supplement: Supplementary file 1 — Supplementary file1 (DOCX 117 KB) [file 12149_2025_2079_MOESM1_ESM.docx]

**Pharmacokinetics and Dosimetry of [^177^Lu]Lu-PSMA-617 and [^68^Ga]Ga-PSMA-11 in Japanese Patients With PSMA-positive mCRPC**

**Author names:** Shoko Takano,^1^ Anri Inaki,^2^ Kenji Hirata,^3^ Richard B. Sparks,^4^ Masahiko Sato,^5^ Satoshi Nomura,^6^ Toru Hattori,^7^ Hiroya Kambara,^7^ Quyen Nguyen,^8^ Tohru Shiga,^9^ Seigo Kinuya,^10^ Makoto Hosono^11^

**Supplementary Data**

**Material and methods**

**Eligibility criteria**

**General Criteria:**

- - Castrate level of serum/plasma testosterone (<50 ng/dL or <1.7 nmol/L).
  - Progressive mCRPC can be based on one of the following: Serum PSA progression (2 consecutive increases over a previous reference value ≥ 1 week apart, with a minimal start value of 2.0 ng/mL or 1.0 ng/mL if PSA is the only indication of progression), soft-tissue progression (≥ 20% increase in the sum of the diameter of all target lesions or new lesions), or progression of bone disease (≥ 2 new lesions on a bone scan).
  - Previous histological, pathological, and/or cytological confirmation of prostate cancer.
  - No previous treatment with Strontium-89, Samarium-153, Rhenium-186, Rhenium-188, Radium-223, or hemi-body irradiation within 6 months of enrollment.
  - No known hypersensitivity to the components of [^177^Lu]Lu-PSMA-617, [^68^Ga]Ga-PSMA-11, or similar drugs.
  - No concurrent cytotoxic chemotherapy, immunotherapy, radioligand therapy, PARP inhibitors, biological therapy, AKT inhibitors, or investigational therapy.
  - No history of CNS metastases unless treated and neurologically stable, asymptomatic, and not requiring corticosteroids for neurologic integrity.
  - No symptomatic cord compression or clinical/radiologic findings indicative of impending cord compression.

**Post-Taxane Population:**

- - Received at least one ARPI in either the hormone-sensitive/castrate-resistant or non-metastatic/metastatic prostate cancer setting.
  - Treated with at least 1, but no more than 2 prior taxane regimens (minimum exposure of 2 cycles per regimen). Eligible if deemed unsuitable for a second regimen by their physician.
  - ECOG performance status of 0 to 2.
  - No systemic anti-cancer therapy (excluding ARPI) within 28 days prior to enrollment.

**Pre-Taxane Population:**

- - Progressed only once on prior second-generation ARPI and a candidate for a change in ARPI as assessed by the treating physician.
  - Second-generation ARPI must be the most recent therapy received.
  - ECOG performance status of 0 to 1.
  - No prior treatment with PARP inhibitors, cytotoxic chemotherapy for castration-resistant or castrate-sensitive prostate cancer, immunotherapy, or biological therapy (excluding ARPI).

**Dosimetry and Pharmacokinetic (PK) Assessments for [^68^Ga]Ga-PSMA-11**

Regions of interest (ROIs) to create volume of interest (VOI) were constructed on the PET images for organs and tissues showing specific uptake of activity mainly the stomach wall, gastrointestinal (GI) total contents, heart wall, kidneys, lacrimal glands, liver lumbar spine, pancreas, prostate, parotid salivary glands, submandibular salivary glands, spleen, testes, thyroid, and whole body. The PET VOI activity concentration and volume statistics for all VOIs were determined using the FDA cleared OSIRIX MD software and/or Hermes Affinity Viewer. Resultant kinetic data were modelled to determine normalized number of disintegrations (NNDs) in all organs and tissues analyzed, and these NNDs were used with the medical internal radiation dose (MIRD)/Radiation Dose Assessment Resource (RADAR) method for internal dosimetry as implemented in the Food and Drug Administration (FDA) cleared Organ Level Internal Dose Assessment/Exponential Software Modeling (OLINDA/EXM) software to produce radiation exposure estimates. Lacrimal gland dosimetry not included in OLINDA was determined by the standard MIRD/RADAR methodology using spherical S-values. A urinary bladder voiding interval of 3.5 hours was utilized.

Total volume of urine voided, the activity assay results from an aliquot sample, and the dates and times that the assays were performed were recorded. These data were utilized to estimate urinary excretion. Organ time activity curves were derived directly from the PET VOIs activity concentrations. The human alimentary model, and urinary voiding bladder model (with a voiding interval of 3.5 h) as implemented in OLINDA were used.

The original blood concentrations of [^68^Ga]Ga-PSMA-11 were decay-corrected to the time of injection and converted to units of percent injected activity per liter (%IA/L). The %IA as a function of time was determined for the quantified organs.

**Dosimetry and Pharmacokinetic Assessments for [^177^Lu]Lu-PSMA-617**

Total volume of urine voided, the activity assay results from an aliquot sample, and the dates and times that the assays were performed were recorded. These data were utilized to estimate urinary excretion. Planar ROI data and SPECT VOI data were quantified to determine the kinetic data as per the Medical Internal Radiation Dose (MIRD) Pamphlet 16 methodology in the organ/tissues, and whole body [1].

Radioactivity in blood and urine for both [^68^Ga]Ga-PSMA-11 and [^177^Lu]Lu-PSMA-617 was determined using a properly calibrated gamma counter with both the lower limit of quantification (LLOQ) and upper limit of quantification (ULOQ) recorded at the investigational sites. For the red marrow dosimetry, a red marrow to blood concentration ratio (RMBLR) of unity was assumed.

ROIs were constructed on the whole-body conjugate planar images for the brain, GI tract, heart, kidneys, lacrimal glands, liver, lungs, salivary glands, spleen, image reference standard, thyroid, urinary bladder, and whole body.

ROI count statistics were quantified to determine bio-kinetic (time-activity) data in the organs and tissues. Red marrow activity was estimated from blood sample assays. Kinetic data were modelled to determine NNDs, which were used with the RADAR/MIRD method for internal dosimetry as implemented in the FDA cleared OLINDA software to produce radiation exposure estimates [2,3]. The lacrimal gland dosimetry is not included in OLINDA, and it was determined using the standard MIRD/RADAR methodology.

For kidneys, liver, and spleen, kinetic data utilized in the dosimetry analysis are based on the SPECT results. Within the OLINDA software the user selects the nuclide, the radiation transport phantom, and enters the NNDs (residence times) for all source organs. OLINDA/EXM multiples these S-values by the NNDs to produce final organ and tissue dose estimates. The human alimentary model, and urinary voiding bladder model (with a voiding interval of 3.5 hours) as implemented in OLINDA were used. Each lacrimal gland was assumed to be a 0.7 g sphere and the S-value for the sphere was determined using non-linear regression of sphere S-values for ^177^Lu obtained from the OLINDA sphere model. The lacrimal dosimetry results represent self-dose only.

**External Radiation Exposure Measurement to Medical Personnel**

The medical personnel wore radiation protection suit with dosimeters placed both inside and outside the suit. The external radiation exposure is measured in a) medical personnel before and after administration of [^177^Lu]Lu-PSMA-617; b) medical personnel accompanying the patient; c) medical personnel when entering a hospital room. For medical personnel accompanying the patient, in case of use of the special measures room, the patient needed to move from the nuclear medicine examination room where [^177^Lu]Lu-PSMA-617 was administered to the patient’s inpatient room. The radiation exposure of HCPs who accompanied the patient during the move from the nuclear medicine examination room to the patient’s inpatient room was measured. Supplementary **Table 1** summarizes the external radiation exposure measurements in various scenarios during [^177^Lu]Lu-PSMA-617 administration.

**Measurement of External Radiation Exposure in the Patient’s Inpatient Room and the Adjacent Room**

The external radiation dose rate (µSv/h) in the patient’s inpatient room was measured at a distance of 1 meter (m) from the vertical and horizontal center of the bed (while the patient is lying in bed) in the patient’s inpatient room and a height of 1 m from the floor. As it is difficult to directly assess the dose of other hospitalized patients, the dose rate in the patient’s room and the dose rate in the adjacent room were measured after administration, by measuring the dose rate in the adjacent room from the boundary wall towards the patient’s bed, and the distance was recorded. The external radiation dose rate in the adjacent room was measured for only 2 patients in special measures room, as 4 patients did not use special measures room (instead radiation treatment room was used by them) and in remaining 2 patients, lead shielding was installed on both neighboring walls of the patient’s room; hence, the dose rate was not measured. These were measured using an ionization chamber or a NaI (Tl) scintillation counter. The first measurement was taken 4 (± 2) hours after the end of the [^177^Lu]Lu-PSMA-617 administration on day 1. From day 2, measurements were taken every 24 (± 4) hours until discharge.

**Airborne Radioactivity Concentration in Patient’s Room**

The measurement of airborne radioactivity concentration in the patient’s inpatient room was performed by air sampling at two places: the center of the room and bedside. A low-volume air sampler was used for air sampling, and a filter paper for dust monitoring (HE-40T, Advantec, Retention efficiency: >99.7%) combined with cellulose fiber and glass fiber was used as a collector. The flow volume was 780 - 900 L and the flow rate were set at about 30 L/min, and the collection efficiency was set at 100% [4]. For the measurement of total gamma radioactivity, a NaI (Tl) scintillation counter (JDC-R74, Hitachi, Ltd., Japan) was used.

**Measurement of External Radiation Exposure to Family Member Living with the Patient**

Before study participation, patients and their families were informed regarding precautions for patients and their family (shown in the supplemental section) including keeping maximum feasible distance from patient and sleeping in a different room for 1 week after administration to reduce radiation exposure. The external radiation exposure of the person accompanying the patient from site to home and the family member living with the patient, until the patient goes home after discharge was measured using a semiconductor personal dosimeter (RPL Dosemeter, Chiyoda Technol Corporation, Japan) [5].

**Precautions for Patients and Their Families in the Week Following Administration of
[^177^Lu]Lu-PSMA-617 (1st Week After Administration of [^177^Lu]Lu-PSMA-617)**

**Precautions regarding routine activities:**

- - 1. If a patient loses blood, that blood will be wiped up with toilet paper and flushed down the toilet.
    2. When there is any potential for coming into contact with a patient’s urine or feces or with clothing contaminated by a patient’s urine or feces, disposable gloves such as rubber gloves will be worn.
    3. When a patient’s bodily fluids such as blood come into contact with the hands or skin, the contaminated site will be immediately washed with soap.
    4. Sexual intercourse is prohibited.
    5. Individuals living with a patient should be separated from the patient to the extent possible. A distance of at least 1 meter should be maintained. When together for a prolonged period, a distance of ≥2 meter should be maintained. Contact with infants and pregnant women will be minimized.
    6. Sleeping with someone else in the same bed will be avoided. A patient should sleep at least 2 meter away. If possible, the patient should sleep in a separate room.
    7. The patient will bathe last. After bathing, the bathtub will be cleaned and washed with a brush and cleaning agent.
    8. Outings in public settings (e.g. public transportation, supermarket, shopping centers, movie theaters, restaurants, and sport venues) should be avoided to the extent possible. When traveling by public transportation, the patient should be separate from other travelers (a distance of ≥1 meter). Travel time in the same vehicle will be reduced so that the patient does not spend >6 hours on the same form of public transportation. When traveling by taxi, the patient will sit as far away from the driver as possible and travel time with the same driver will be reduced.

**Precautions regarding handling laundry:**

- - 1. Clothing worn by a patient administered the labelled somatostatin analogue will be washed separately from the clothing of other individuals, and not at the same time. In addition, bed linens and undergarments soiled with blood or urine will be prepared for washing.

**Precautions regarding urination, defecation, or vomiting:**

- - 1. Patients will urinate while seated.
    2. When feces or urine soil the toilet or floor, that material will be wiped up with toilet paper and flushed down the toilet.
    3. A toilet will be flushed about two times after use.
    4. Hands will be washed and cleaned with soap after urination or defecation.
    5. Hands and skin that come into contact with a patient’s bodily fluids (e.g. blood), excreta, or vomitus will be cleaned and washed with soap.

The family member was instructed to always wear or bring the personal glass dosimeter with them to measure their personal external radiation exposure. The provided dosimeter was collected or returned on day 15 of the [^177^Lu]Lu-PSMA-617 administration. An additional dosimeter for background measurements was provided to the family member, placed far from the patient, such as at the entrance or storage room, and collected on day 15 of the cycle. The family member living with patient also entered records of their activities when they approach the patient (<1 m) and answered a

**Results**

**External Radiation Exposure to the Medical Personnel from Preparation Before Administration to Completion of Administration of [^177^Lu]Lu-PSMA-617**

The time from preparation start to administration end for this outlier was 155 minutes, which is longer than the mean time (84.5 minutes). This medical personnel did not wear the protection suit, hence no data available with the suit. It might have been possible to decrease the radiation exposure if the personnel wore the protection suit (**Table 3; Supplementary Figure 1**)

**Exposure to the Medical Personnel When Entering a Hospital Room**

The exposure within 1-hour post-administration was slightly higher than that after 4 hours in all but one medical personnel (8.6 μSv at 4 to 24 hours post administration outside the radiation protection suit); however the dose was effectively reduced by wearing the suit (**Supplementary Table 6**).

**External Radiation Dose Rate in the Adjacent Room of Patients’ Room**

The external radiation dose rate in the adjacent room was measured for only 2 patients in special measures room, as 4 patients did not use special measures room (instead radiation treatment room was used by them) and in remaining 2 patients, lead shielding was installed on both neighboring walls of the patient’s room; hence, the dose rate was not measured.

**Family Member Radiation Exposure**

In 1 patients’ family member with 60 μSv radiation exposure, hospitalization was prolonged for the patient due to a serious adverse event (SAE) that occurred on day 1. The SAE subsequently improved and the patient was discharged on day 7. Patients may be in poor condition, hence requiring physical support closer to them than usual, which could be potentially contributing to the higher radiation dose observed. With respect to compliance with the use of a glass badge dosimeter by the patient’s family, all of them always used it except for 1 day (5 days after discharge) in 1 patient**.** The hospital stay varied extensively from 2 to 7 days (1 to 6 nights) after the administration of [^177^Lu]Lu-PSMA-617.

**Figures and Tables**

**Supplementary Fig. 1:** Plot of external radiation exposure to medical personnel from preparation to completion of administration of [^177^Lu]Lu-PSMA-617


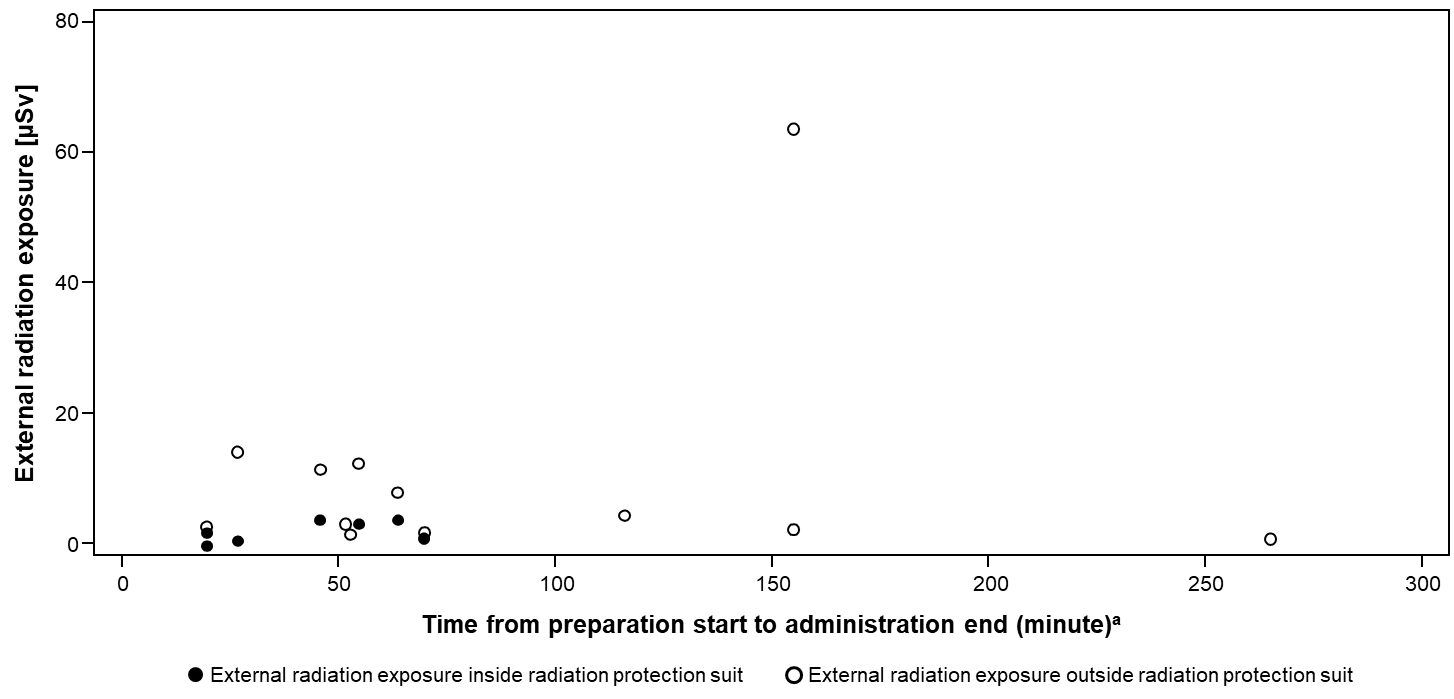

^a^Time from preparation start to administration end = Administration end time − Preparation start time.

^177^Lu, lutetium-177; μSv, microsievert; PSMA, prostate-specific membrane antigen

**Supplementary Table 1:** Measurement of external radiation exposure in medical personnel during
[^177^Lu]Lu-PSMA-617 administration

| External radiation exposure measurement in personnel | Activity | Measurement | Dosimeter position | Location |
| --- | --- | --- | --- | --- |
| Medical personnel involved in the administration of [^177^Lu]Lu-PSMA-617 | From preparation before administration to completion of administration of  [^177^Lu]Lu-PSMA-617 | Using semiconductor personal dosimeter. | Inside and outside the radiation protection suit | Both types of inpatient rooms (radiation treatment room or special measures room) |
| Medical personnel accompanying the patient | After the administration of [^177^Lu]Lu-PSMA-617 when the patient was moved to the inpatient room | Using semiconductor personal dosimeter. | Inside and outside the radiation protection suit | Nuclear medicine examination room (radiation-controlled area), and the patient moved to the special measures room |
| Medical personnel when entering a hospital room | After  [^177^Lu]Lu-PSMA-617 administration and the transfer of the patient to the inpatient room | Using semiconductor personal dosimeter. | Inside and outside the radiation protection suit | Inpatient room (special measures room) |
| ^177^Lu, lutetium-177; PSMA, prostate specific membrane antigen | | | | |

**Supplementary Table 2:** Summary of [^68^Ga]Ga-PSMA-11 whole blood concentration (%IA/L) vs. time

| **Time (hr)** | | | | | | | |
| --- | --- | --- | --- | --- | --- | --- | --- |
|  | **0.083** | **0.25** | **0.5** | **0.75** | **1.42** | **2.92** | **4.08** |
| N | 3 | 3 | 3 | 3 | 3 | 3 | 3 |
| Mean | 6.31 | 4.09 | 3.17 | 2.61 | 1.97 | 1.50 | 1.62 |
| SD | 1.50 | 1.26 | 0.864 | 0.644 | 0.664 | 0.520 | 0.613 |
| %CV | 23.9 | 30.9 | 27.3 | 24.7 | 33.8 | 34.7 | 37.9 |
| Geometric Mean | 6.18 | 3.95 | 3.08 | 2.55 | 1.88 | 1.43 | 1.55 |
| Geometric %CV | 26.1 | 34.0 | 31.0 | 27.4 | 39.4 | 37.5 | 36.6 |
| Note: Sample concentrations were corrected for radioactive decay. ^68^Ga, gallium-68; CV, coefficient of variance; hr, hour; PSMA, prostate specific membrane antigen; SD, standard deviation | | | | | | | |

**Supplementary Table 3:** Summary of [^177^Lu]Lu-PSMA-617 whole blood concentration (ng/mL) vs. time

| **Time (hr)** | | | | | | | | | | |
| --- | --- | --- | --- | --- | --- | --- | --- | --- | --- | --- |
|  | **Pre-dose** | **0** | **0.33** | **1** | **2** | **4** | **24** | **48** | **72** | **120** |
| N | 3 | 3 | 3 | 3 | 3 | 3 | 3 | 3 | 3 | 3 |
| Mean | 0.00 | 13.5 | 7.75 | 5.35 | 4.06 | 2.89 | 0.380 | 0.0777 | 0.0345 | 0.0142 |
| SD | NA | 3.06 | 1.82 | 1.30 | 0.908 | 0.818 | 0.213 | 0.0395 | 0.0204 | 0.00760 |
| %CV | NA | 22.6 | 23.5 | 24.2 | 22.3 | 28.3 | 56.1 | 50.8 | 59.1 | 53.7 |
| Geometric Mean | NA | 13.3 | 7.60 | 5.24 | 3.99 | 2.81 | 0.345 | 0.0715 | 0.0309 | 0.0129 |
| Geometric %CV | NA | 24.8 | 24.9 | 25.6 | 24.6 | 31.5 | 56.2 | 52.1 | 61.6 | 57.9 |
| Note: Sample concentrations were corrected for radioactive decay. ^177^Lu, lutetium-177; CV, coefficient of variation; hr, hour; PSMA, prostate specific membrane antigen; SD, standard deviation | | | | | | | | | | |

**Supplementary Table 4:** Absorbed dose estimates of [^68^Ga]Ga-PSMA-11 (mGy/MBq)

|  | **Patients** | | |  |
| --- | --- | --- | --- | --- |
| **Source Organs** | **Patient 1** | **Patient 2** | **Patient 3** | **Mean ± SD** |
| Adrenals | 0.052 | 0.050 | 0.038 | 0.046 ± 0.0074 |
| Brain | 0.0087 | 0.0053 | 0.010 | 0.0079 ± 0.0023 |
| Esophagus | 0.013 | 0.017 | 0.012 | 0.014 ± 0.0024 |
| Eyes | 0.0086 | 0.0053 | 0.010 | 0.0078 ± 0.0022 |
| Gallbladder Wall | 0.021 | 0.058 | 0.018 | 0.032 ± 0.023 |
| Left colon | 0.027 | 0.020 | 0.025 | 0.024 ± 0.0038 |
| Small Intestine | 0.073 | 0.042 | 0.063 | 0.059 ± 0.016 |
| Stomach Wall | 0.047 | 0.026 | 0.032 | 0.035 ± 0.011 |
| Right colon | 0.064 | 0.043 | 0.056 | 0.054 ± 0.010 |
| Rectum | 0.017 | 0.0083 | 0.013 | 0.013 ± 0.0042 |
| Heart Wall | 0.054 | 0.045 | 0.036 | 0.045 ± 0.0089 |
| Kidneys | 0.34 | 0.068 | 0.27 | 0.23 ± 0.14 |
| Lacrimal Glands | 0.13 | 0.024 | 0.24 | 0.13 ± 0.11 |
| Liver | 0.072 | 0.39 | 0.037 | 0.17 ± 0.19 |
| Lungs | 0.012 | 0.017 | 0.012 | 0.013 ± 0.0030 |
| Pancreas | 0.018 | 0.023 | 0.016 | 0.019 ± 0.0038 |
| Prostate | 0.029 | 0.032 | 0.014 | 0.025 ± 0.0097 |
| Salivary Glands | 0.14 | 0.042 | 0.17 | 0.12 ± 0.068 |
| Red Marrow | 0.019 | 0.014 | 0.018 | 0.017 ± 0.0023 |
| Osteogenic Cells | 0.015 | 0.011 | 0.014 | 0.013 ± 0.0023 |
| Spleen | 0.055 | 0.049 | 0.091 | 0.065 ± 0.022 |
| Testes | 0.021 | 0.0062 | 0.011 | 0.013 ± 0.0075 |
| Thymus | 0.012 | 0.011 | 0.012 | 0.011 ± 0.00036 |
| Thyroid | 0.021 | 0.0060 | 0.012 | 0.013 ± 0.0073 |
| Urinary Bladder Wall | 0.24 | 0.031 | 0.032 | 0.10 ± 0.12 |
| Total Body | 0.016 | 0.016 | 0.014 | 0.015 ± 0.0013 |
| **Effective Dose (mSv/MBq)** | **0.036** | **0.032** | **0.023** | **0.03 ± 0.007** |
| ^68^Ga, gallium-68; MBq, megabecquerel; mGy, milligray; mSV, millisievert; PSMA, prostate specific membrane antigen; SD, standard deviation | | | | |

**Supplementary Table 5:** Absorbed dose estimates of [^177^Lu]Lu-PSMA-617 (Gy/GBq)

|  | **Patient number** | | |  | |
| --- | --- | --- | --- | --- | --- |
| **Source Organs** | **Patient 1** | **Patient 2** | **Patient 3** | **Mean ± SD** | |
| Adrenals | 0.021 | 0.025 | 0.017 | 0.021 ± 0.004 | |
| Brain | 0.013 | 0.024 | 0.0093 | 0.015 ± 0.0075 | |
| Esophagus | 0.013 | 0.017 | 0.011 | 0.014 ± 0.0031 | |
| Eyes | 0.012 | 0.015 | 0.0097 | 0.012 ± 0.0028 | |
| Gallbladder Wall | 0.016 | 0.022 | 0.014 | 0.017 ± 0.0041 | |
| Left colon | 0.34 | 0.67 | 0.40 | 0.470 ± 0.17 | |
| Small Intestine | 0.040 | 0.071 | 0.043 | 0.051 ± 0.017 | |
| Stomach Wall | 0.013 | 0.018 | 0.011 | 0.014 ± 0.0032 | |
| Right colon | 0.19 | 0.36 | 0.22 | 0.26 ± 0.095 | |
| Rectum | 0.33 | 0.64 | 0.38 | 0.45 ± 0.17 | |
| Heart Wall | 0.071 | 0.079 | 0.032 | 0.061 ± 0.025 | |
| Kidneys | 0.40 | 0.36 | 0.28 | 0.34 ± 0.059 | |
| Lacrimal Glands | 0.89 | 2.8 | 2.4 | 2.0 ± 1.0 | |
| Liver | 0.092 | 0.11 | 0.093 | 0.100 ± 0.013 | |
| Lungs | 0.038 | 0.044 | 0.039 | 0.040 ± 0.0034 | |
| Pancreas | 0.015 | 0.020 | 0.013 | 0.016 ± 0.0036 | |
| Prostate | 0.015 | 0.020 | 0.013 | 0.016 ± 0.0033 | |
| Salivary Glands | 0.79 | 0.79 | 0.75 | 0.78 ± 0.026 | |
| Red Marrow | 0.029 | 0.042 | 0.029 | 0.033 ± 0.0072 | |
| Osteogenic Cells | 0.024 | 0.031 | 0.021 | 0.025 ± 0.0050 | |
| Spleen | 0.063 | 0.064 | 0.091 | 0.073 ± 0.016 | |
| Testes | 0.012 | 0.015 | 0.010 | 0.012 ± 0.0027 | |
| Thymus | 0.013 | 0.016 | 0.010 | 0.013 ± 0.0029 | |
| Thyroid | 0.046 | 0.057 | 0.045 | 0.049 ± 0.0066 | |
| Urinary Bladder Wall | 0.34 | 0.32 | 0.34 | 0.33 ± 0.012 | |
| Total Body | 0.023 | 0.030 | 0.020 | 0.025 ± 0.0050 | |
| **Effective Dose (mSv/MBq)** | **0.077** | **0.11** | **0.081** | 0.089 ± 0.018 | |
| ^177^Lu, lutetium-177; GBq, gigabecquerel; MBq, megabecquerel; mSV, millisievert; Gy, gray; PSMA, prostate specific membrane antigen; SD, standard deviation | | | | |  |

**Supplementary Table 6:** Table of external radiation exposure of medical personnel when entering a hospital room

|  | External radiation exposure | |  | |
| --- | --- | --- | --- | --- |
| Time from the end of administration to the entry of medical personnel into the hospital room [hr]* | Inside radiation protection suit [μSv] | Outside radiation protection suit [μSv] | | Hospital room stay time of medical personnel [min]^†^ |
| Overall |  |  | |  |
| n | 102 | 170 | | 169 |
| Mean (SD) | 0.076 (0.2204) | 0.23 (0.789) | | 6.4 (16.26) |
| ≤1 |  |  | |  |
| n | 6 | 6 | | 6 |
| Mean (SD) | 0.583 (0.2858) | 1.53 (0.889) | | 12.7 (13.62) |
| <1- ≤4 |  |  | |  |
| n | 13 | 16 | | 16 |
| Mean (SD) | 0.046 (0.0967) | 0.23 (0.235) | | 5.4 (7.91) |
| <4 - ≤24 |  |  | |  |
| n | 28 | 37 | | 37 |
| Mean (SD) | 0.083 (0.3055) | 0.48 (1.518) | | 3.8 (4.48) |
| <24- ≤ 48 |  |  | |  |
| n | 35 | 43 | | 43 |
| Mean (SD) | 0.034 (0.0639) | 0.08 (0.154) | | 5.0 (4.86) |
| <48 - ≤72 |  |  | |  |
| n | 12 | 21 | | 20 |
| Mean (SD) | 0.008 (0.0289) | 0.11 (0.195) | | 5.0 (4.87) |
| 72< |  |  | |  |
| n | 8 | 47 | | 47 |
| Mean (SD) | 0.000 (0.0000) | 0.05 (0.091) | | 9.7 (29.20) |
| *Time from the end of administration to the entry of medical personnel into the hospital room = Entry time of medical personnel into the hospital room − Administration end time ^†^Hospital room stay time of medical personnel = Time of leaving the patient's room − Time of entering the patient's room If the calculated value is less than 1 minute, it is summarized as 1 minute.  μSv, microsievert; hr, hour; min, minute; SD, standard deviation | | | | |

**Supplementary Table 7:** External radiation dose rate in patient's room

|  | Patient's room [μSv/h] |
| --- | --- |
| Day 1 (4 h) |  |
| n | 8 |
| Mean (SD) | 20.625 (7.8029) |
| Day 2 (24 h) |  |
| n | 9 |
| Mean (SD) | 7.000 (2.2820) |
| Day 3 (48 h) |  |
| n | 7 |
| Mean (SD) | 4.297 (1.1631) |
| Day 4 (72 h) |  |
| N | 3 |
| Mean (SD) | 3.467 (0.3786) |
| Day 5 (96 h) |  |
| N | 2 |
| Mean (SD) | 2.250 (0.0707) |
| Day 6 (120 h) |  |
| n | 2 |
| Mean (SD) | 2.015 (0.0212) |
| Day 7 (144 h) |  |
| n | 1 |
| Mean (SD) | 1.700 (NE) |
| Day 8 (168 h) |  |
| n | 1 |
| Mean (SD) | 0.760 (NE) |
| If a patient is measured in multiple cycles, all measurements are used for the summary and the 'n' is counted as more than one for the patient. | |
| μSv, microsievert; h, hour; NE, not estimable; SD, standard deviation | |

**References**

**1.** Siegel JAT SR, Stubbs JB, Stabin MG, Hays MT et al. MIRD pamphlet no. 16: Techniques for quantitative radiopharmaceutical biodistribution data acquisition and analysis for use in human radiation dose estimates. *J Nucl Med.* 1999;40:37S-61S. (**[PMID: 10025848]**)

**2.** Stabin MGS RB, Crowe E. OLINDA/EXM: The Second-Generation Personal Computer Software for Internal Dose Assessment in Nuclear Medicine. *J Nucl Med.* 2005;46:1023-1027.

**3.** Stabin M, Farmer, A. OLINDA/EXM 2.0: The new generation dosimetry modeling code. *J Nucl Med.* 2012;53: 585.

**4.** Yamamoto H KM. Radiation monitoring of the workplace (VI). Air monitoring. (2) Monitoring of radioactive particulate material concentration in air (author's transl). *Radioisotopes.* 1977;26:432-442. (**[PMID: 578968]**)

**5.** RPL Dosemeter for Individual Monitoring. https://www.c-technol.co.jp/en/pdf/Personal-Dosimetry-System_5th_Edition.pdf. Accessed 25-11-2024, 2024.
